# The sword in the wall: managing biliary stents embedded in the duodenal wall following ERCP for biliary strictures after liver transplantation in an adult and a pediatric patient

**DOI:** 10.1055/a-2329-2042

**Published:** 2024-06-07

**Authors:** Giacomo Emanuele Maria Rizzo, Ilaria Tarantino, Lucio Carrozza, Dario Ligresti, Gabriele Rancatore, Marco Giacchetto, Mario Traina

**Affiliations:** 1 18326 Endoscopy Service, Department of Diagnostic and Therapeutic Services, ISMETT, Palermo, Italy; 2 18998 Department of Precision Medicine in Medical, Surgical and Critical Care (Me.Pre.C.C.), University of Palermo, Palermo, Italy; 3 18326 Hepatology Section, Department of the Treatment and Study of Abdominal Diseases and Abdominal Transplantation, ISMETT, Palermo, Italy

We present the minimally invasive management of a rare delayed complication that can occur after biliary stenting for benign biliary anastomotic stricture (BBAS) after orthotopic liver transplantation (OLT).

**Fig. 1:**
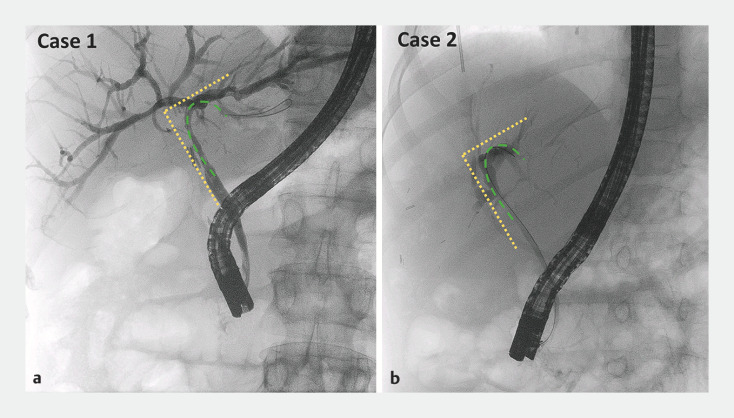
Radiologic views of the angled biliary duct upstream of the anastomotic biliary stricture, with similar appearances in: **a** case #1; **b** case #2.

Case #1 is that of a 69-year-old man with a history of OLT for hepatocellular carcinoma and cirrhosis related to hepatitis C virus. One year after OLT, he developed a BBAS that was managed with endoscopic retrograde cholangiopancreatography (ERCP), which confirmed a stricture in the anastomotic site involving the proximal/hilar site of the common bile duct (CBD), creating an angle of about 90° with the right and left biliary ducts Fig. 1 **a** ). Multistenting treatment was begun through the insertion of a plastic biliary stent (10 Fr × 12 cm) with the distal side following the angled shape of the biliary duct to bridge the stricture.

**Fig. 2:**
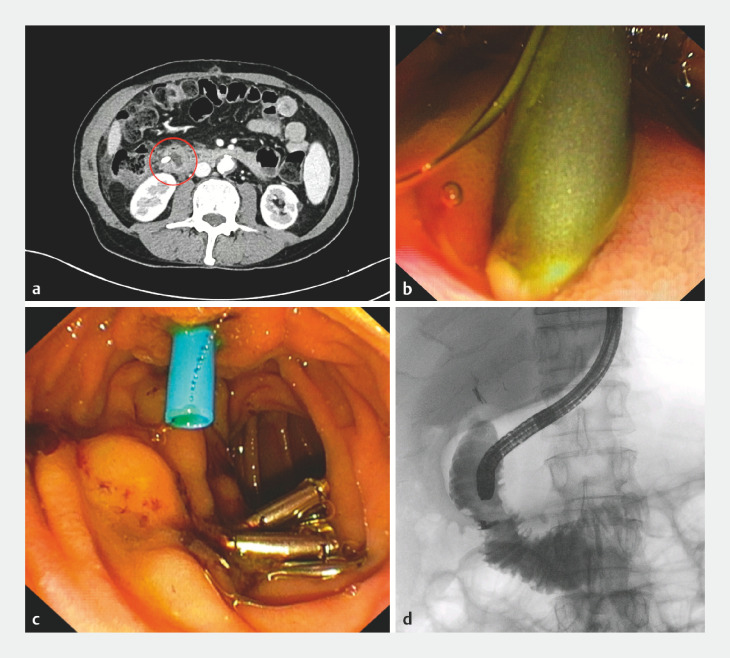
Images of case #1 showing: **a** on a computed tomography scan, the proximal side of the biliary stent crossing the duodenal wall (red circle); **b, c** on endoscopic views: **b** the distal side of the biliary plastic stent embedded into the duodenal wall; **c** clips applied to the defect and a new biliary stent passing through the major papilla; **d** on fluoroscopy, no leakage of intraluminal contrast or other signs of perforation after clip placement.

After two months, the patient developed fever, abdominal pain, jaundice, and itching, so a computed tomography scan was performed, which showed the proximal side of the plastic stent was embedded in the duodenal wall ( Fig. 2 **a** ). The patient underwent a second ERCP, which showed the proximal side of the stent to be completely trapped in the wall of the second part of the duodenum, opposite to the major papilla, with the plastic wing totally incorporated into the duodenal wall ( Fig. 2 **b** ). Therefore, even though we were worried about perforation, we extracted the stent with forceps, gently applying a slight traction with the scope under radiologic visualization ( Video 1 ). Subsequent evaluation of the duodenal wall showed an ulcerated area with pigmented hematin at its base, but no endoscopic or radiologic signs of perforation, so we closed the defect with four through-the-scope metal clips. No signs of extraluminal diffusion were seen after intraluminal contrast injection, so we successfully completed the ERCP with insertion of a shorter plastic stent to continue the treatment of the BBAS ( Fig. 2 **c, d** ).

Management of an adult and a pediatric patient with a delayed complication of stenting for benign biliary stricture at the biliary anastomosis after liver transplantation, with the biliary stent embedding into the duodenal wall and being endoscopically removed.Video 1

**Fig. 3:**
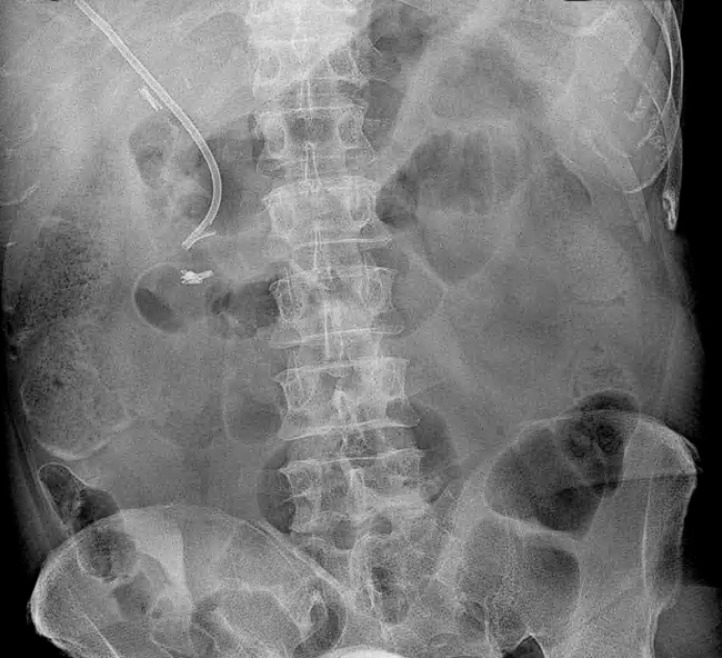
Abdominal radiograph taken after 12 hours showing no signs of perforation.

The patient fully recovered, with no signs of duodenal perforation, as confirmed by an abdominal radiograph after 12 hours ( Fig. 3 ), so he started feeding and was discharged 72 hours after the procedure. At follow-up after 3 months, he appeared well and the multistenting treatment was continued.

**Fig. 4:**
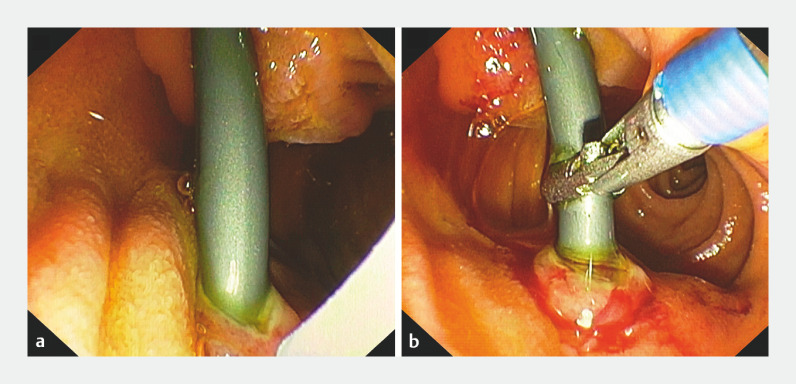
Endoscopic views showing: **a** the embedded stent; **b** forceps being used to remove the embedded stent.

**Fig. 5:**
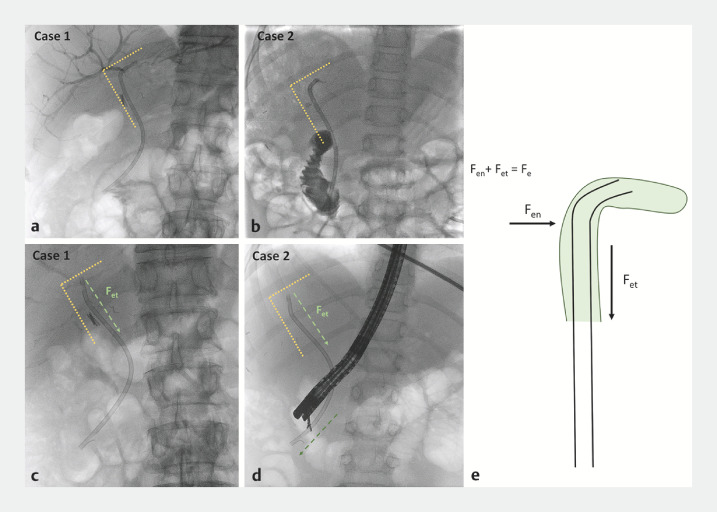
Radiologic views of the biliary stents placed upstream to the biliary anastomotic stricture for: **a, c** case #1; **b, d** case #2; **a, b** with the proximal sides creating angles (about 90°) into the left hepatic duct; **c, d** 3 months after the first ERCP, with straightened and descended biliary stents. **e** A schematic representation of the two components of the elastic force of the stent: the normal elastic force (F _en_ ) and the tangential elastic force (F _et_ ), with F _et_ being the one that makes the tube retract, while F _en_ makes the tube straighten up.

A similar complication occurred in case #2, a boy of 11 years of age with a BBAS after living-donor liver transplantation ( Fig. 1 **b** ). He underwent ERCP for the insertion of a second plastic biliary stent, but endoscopic view in the second part of the duodenum showed the plastic stent was embedded in the lateral duodenal wall ( Fig. 4 **a** ). Even in this case, he had a proximal stricture involving the hilar part of the biliary system, where the first plastic stent was positioned, with an angled distal site upstream of the stricture. We removed the stent with forceps ( Fig. 4 **b** ) and closed the mucosal defect with two metal clips, with no evidence of extraluminal contrast diffusion at fluoroscopy. In this second case as well, the angle of the proximal side of the stent was acute, suggesting a vector distribution of the force pushing the stent downstream, losing its location and creating continuous pressure against the contralateral duodenal wall until the stent embedded within it ( Fig. 5 ).

Our theoretical explanation of this complication takes into consideration different forces: the elasticity of the stent, which depends on the material (plastic) and generates the elastic force (a restoring force), and the force of vincular reaction, which is generated by the biliary duct and works against the stent. The elastic force is directly proportional to the deformation produced and is opposite in direction to the one causing the deformation. The relationship between the elastic force and the deformation is known as Hooke’s law (F _e_ = − *k* x; where F _e_ = the elastic force, *k* = the elastic constant, x = the displacement from the equilibrium state). Moreover, the elastic force can be broken down into two components: one perpendicular to the duct, called the normal elastic force (F _en_ ), and one parallel to the duct, called the tangential elastic force (F _et_ ). The F _et_ is the one that makes the tube retract, while the F _en_ is the one that makes the tube straighten up ( Fig. 5 **e** ).

Endoscopy_UCTN_Code_TTT_1AR_2AZ

